# Unexplained Graft Dysfunction after Heart Transplantation—Role of Novel Molecular Expression Test Score and QTc-Interval: A Case Report

**DOI:** 10.4061/2010/230810

**Published:** 2010-06-22

**Authors:** Khurram Shahzad, Martin Cadeiras, Kotaro Arai, Dmitry Abramov, Elizabeth Burke, Mario C. Deng

**Affiliations:** College of Physicians and Surgeons, Columbia University, New York, NY 10032, USA

## Abstract

In the current era of immunosuppressive medications there is increased observed incidence of graft dysfunction in the absence of known histological criteria of rejection after heart transplantation. A noninvasive molecular expression diagnostic test was developed and validated to rule out histological acute cellular rejection. In this paper we present for the first time, longitudinal pattern of changes in this novel diagnostic test score along with QTc-interval in a patient who was admitted with unexplained graft dysfunction. Patient presented with graft failure with negative findings on all known criteria of rejection including acute cellular rejection, antibody mediated rejection and cardiac allograft vasculopathy. The molecular expression test score showed gradual increase and QTc-interval showed gradual prolongation with the gradual decline in graft function. This paper exemplifies that in patients presenting with unexplained graft dysfunction, GEP test score and QTc-interval correlate with the changes in the graft function.

## 1. Introduction

Cardiac allograft rejection after heart transplantation (HTx) can involve both cellular and antibody mediated immune injury to the allograft. Hyperacute rejection is an antibody-mediated process which occurs typically minutes to hours after transplantation. Acute cellular rejection (ACR) is a T-lymphocyte mediated process that occurs from the first week to years after HTx and is the major cause of graft loss early after HTx although its incidence declines after first year [1]. ACR is diagnosed by endomyocardial biopsy (EMB) and graded by degree of lymphocyte infiltration into myocardium according to International Society of Heart and Lung Transplantation (ISHLT) guidelines [2, 3]. Acute antibody mediated rejection (AMR) was reported to be present in 2% to 20% of EMBs after heart transplantation [1, 4–8]. It is classically demonstrated by linear deposition of immunoglobulins and complement splitting products such as C4d in the vascular endothelium [8]. Cardiac allograft vasculopathy (CAV) is one of the major causes of late graft dysfunction (GD). It is diagnosed by annual coronary angiography or intravascular ultrasound (IVUS). Historically invasive EMB was only indicated based on the clinical suspicion of rejection assessed by electrocardiographic and hemodynamic parameters. Recently, using postgenome-era high-throughput transcriptomic technology, a novel non-invasive molecular expression diagnostic test was developed and validated against EMB based criteria of ACR [9]. This peripheral blood mononuclear cell gene expression profiling (GEP) test with a score <34 has very high negative predictive value of 99.6% to rule out moderate/severe (≥2R/3A) ACR [10]. With the advancement in the immunosuppressive medications in the transplantation medicine, there is increased observed incidence of unexplained graft dysfunction in HTx patients. In this paper we present for the first time the role of longitudinal patterns of this novel molecular expression test and QTc-interval in a patient admitted with unexplained graft dysfunction.

## 2. Case Presentation

A 59-year-old gentleman with a history of ischemic cardiomyopathy underwent orthotopic heart transplantation in our center on October 10, 2005. The donor was a 36-year-old male matched for body size with negative serologies for Cytomegalovirus and Toxoplasma. Postoperatively, his overall course was stable and followed with EMB without significant history of ACR. During the first 6 months rejection monitoring was EMB based. After the 9th month, we transitioned to non-invasive GEP test based monitoring [9] on the basis of consensus recommendations [10]. In the overlap phase (concomitant invasive and non-invasive monitoring), an EMB on June 6, 2006, showed absence of rejection (Quilty lesions) with a GEP test score of 26, right atrial pressure of 5 mmHg, pulmonary artery pressures of 31 mmHg systolic, 11 mmHg diastolic, and a mean of 22, pulmonary capillary wedge pressure of 13, an O_2_ mixed venous saturation of 75%, and a cardiac output (CO) by thermodilution of 5.86 l/min (Cardiac Index 2.55 l/min/sqm). A follow up biopsy on January 11, 2007 showed ISHLT grade 0 with a GEP score of 28 (Table 1). From that time on, the patient was followed non-invasively with the GEP test and echocardiography and had a clinically stable course until January 2008. A follow up echocardiogram obtained on January 17, 2008 showed reduction in left ventricular ejection fraction (LVEF) from normal to 43%. The patient's immunosuppressive regimen at that moment included cyclosporine (CsA) 100 mg twice a day, mycophenolate 250 mg twice a day and prednisone 5 mg daily. In addition, the patient was also receiving pravastatin 20 mg, aspirin 81 mg, doxazosin 1 mg, amlodipine 2.5 mg and carvedilol 25 mg. 

On January 29 2008, the patient presented to our clinic with atypical chest pain, shortness of breath, and hemoptysis. His Initial evaluation revealed blood pressure of 120/80 mmHg, heart rate 92 bpm regular, temperature 98.7, and respiratory rate of 22. His NYHA functional class was III-IV, his lung auscultation was remarkable for bilateral crackles, and his heart auscultation was positive for an S3 gallop. The abdomen was soft without organomegaly and the patient had no lower extremity edema. A chest X-ray showed new extensive right perihilar disease. An EKG showed no acute changes but his QTc-interval duration was 530 ms. Right heart catheterization and an EMB were done emergently. Hemodynamics showed Pulmonary Artery (S/D/M): 62/33/45, Pulmonary Capillary Wedge (a/v/M): 34/47/39, Right Atrium (a/v/M): 16/14/13, a Mixed Venous O_2_ Saturation of 51% and CO by thermodilution of 3.20 l/min (Cardiac Index = 1.38 l/min/sqm). 

**Figure 1:**
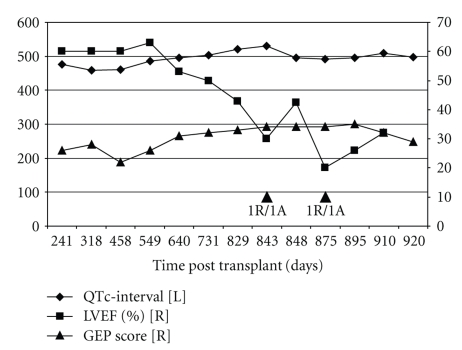
Longitudinal post-HTx changes in the GEP test score, QTc-interval, and Left Ventricular Ejection Fraction. L = left axis; R = right axis; LVEF = left ventricular ejection fraction; GEP = gene expression profiling test score; QTc = corrected QT interval.

The review of the patient posttransplant management during the last year showed a progressive declination of the graft function within the range of normality values (Table 1), prolongation of the QTc-interval on the EKG, and progressive increase in his GEP scores (Figure 1). After the clinically indicated EMB, patient was treated with intravenous steroids under the presumed diagnosis of ACR. The EMB showed focal, mild (1A/1R) ACR with nodular endocardial infiltrate (Quilty effect). A C4d immunohistochemical staining was negative for AMR. Respiratory viral panels were negative, and viral inclusions or associated inflammatory response was not found on the EMB. The patient was treated with intravenous (pulse) methyl-prednisolone for three consecutive days (500 milligrams per day) followed by an oral taper of methyl-prednisone in addition to diuretics. A coronary angiogram done on February 05, 2008 showed mild pruning of the distal coronary arteries. A follow-up echocardiogram 4 days after completing treatment showed improvement in regional wall motion abnormality and a significant increase in the LVEF to 40%–45%. The patient was discharged home in a stable condition. On 03/04/2008, a follow-up EMB and right heart catheterization were obtained which showed impaired graft function and 1A/1R biopsy grade with negative C4d staining. The patient was treated with intravenous methyl-prednisolone (500 mg) and a steroid taper. New evaluation with EMB on 04/07/2008 showed absence of lymphocytic infiltrates or C4d deposition. Subsequent evaluation of the graft function by echocardiography showed persistent allograft dysfunction (Table 1).

## 3. Discussion

There were no findings consistent with ACR and AMR in our case. CAV is present in about half of all HTx recipients at 5 years posttransplantation. Although our patient had evidence of mild pruning of the distal coronary vessels on the angiography but there was no significant blockage of any of the main vessels. These angiographic findings were unable to explain the degree of graft dysfunction.

Infectious etiologies are a potential cause for primary or secondary allograft dysfunction during the first years after transplantation with cytomegalovirus as the most common etiologic agent [11]. Repetitive blood cultures and PCR analyses and the absence of intranuclear inclusions in the endomyocardial samples made this possibility very unlikely. Considering the initial presentation of the patient with acute respiratory infiltrates would raise the consideration of a severe systemic acute infection, potentially of viral origin leading to graft loss which has been increasingly reported [12]. An infiltrative myocardial disease of the recipient could be suspected such as amyloidosis or lymphoproliferative disease affecting the heart. Post-HTx lymphoproliferative disease has been reported to have tropism for the transplanted organs and to be a cause of cardiac allograft failure [13]. Neither the clinical findings during this admission nor the biopsy raised this suspicion. 

In the present case, the review of his medical history showed progressive declination of the graft function within the range of normality, with partial improvement after steroid treatment that suggests an acute inflammatory alloimmune mechanism with a negative biopsy. EMB has been used for the last 35 years to screen patients for ACR although it has been limited by invasiveness, complexity, discomfort, complication-proneness, interpretation variability and late detection of rejection. In the current era and state of practice in HTx, the limitations of EMB have been recalled. The likelihood of consensus for the diagnosis of ACR in eyes of highly experienced pathologist is less than expected with 78% best agreement rate for 3A/2R rejection and significantly lower for grades 1B/1R, 1A/1R, and 2/1R. There is 50% downgrading of rejection grade when an independent local pathologist reading is reinterpreted by a core of expert pathologists [14]. 

In original CARGO study, the cutoff of 34 on the GEP test score scale was selected to adjust for the high negative predictive value (NPV) of the test with the intention to rule out ACR. In our patient, GEP test score mostly remained in the lower 30s range. This could be explained by the absence of documented evidence of moderate/severe ACR as defined by histology (≥2R/3A) against which GEP test was developed and validated. Most recently, we have analyzed the variability of the molecular score within and between patients suggesting that not only a cutoff value is important but the time dependent variability of the molecular score may provide a more personalized evaluation [15]. Furthermore, data from CARGO study showed that independently from the cut-off value set for the purpose of the study, as the agreement of rejection diagnosis increased among pathologists, also did the molecular score [9].

In the post implementation clinical experience of GEP test we have demonstrated the relationship of the GEP test score with echocardiographic and electrocardiographic parameters of allograft rejection [16]. In the present case, longitudinal evaluation of the results of the GEP test suggested a progressive increase in the molecular score from a deep quiescent range to an alloactivated status. The time dependent and sustained trend of the molecular test showed a significant variability that followed the progressive left ventricular function deterioration, prolongation of the QTc-interval, and development of acute decompensated graft failure that lead to hospitalization. The presentation of GD without histological evidence of significant ACR, absence of C4d deposition, and other surrogates of rejection suggest the limitation of currently available diagnostic tools. With the failure of currently known histopathological mechanisms to explain the involved immune mechanisms there is need to develop accurate methods beyond histological criteria to detect/predict patients with unexplained graft dysfunction.

## 4. Conclusion

This case report exemplifies that in patients with unexplained graft dysfunction, GEP test score and QTc-interval correlate with the changes in the graft function. These patients are empirically treated as rejection or even infection without an objective proof to support the diagnosis or treatment for these severe life threatening conditions. Furthermore, we use medical treatments that may lead to severe adverse events without exactly knowing which the entity that we are treating is. Further studies are required to better understand the immune mechanisms of unexplained GD and to develop accurate diagnostic tools beyond histological criteria for appropriate treatment interventions.

## Figures and Tables

**Table 1:** Post Transplant management of patient since started on non-invasive rejection surveillance protocol.

Date	Days Post HTx	GEP Score (95% CI)	CsA dose (mg)	CsA level (ng/ml)	MMF dose (mg)	Pred dose (mg)	LVE (%)	Biopsy grade	AMR	RAP	PCWP	PAS	PAD	PAM	CO	MVO_2_ Sat.	QTc
06/08/2006	241	26 (22.8 : 30.0)	350	236	250	5	60	0		5	11	34	10	16	5.86	74	477
08/24/2006	318	28 (25.4 : 31.8)	350	179	500	5		0		5	13	31	11	22	4.16	82	459
01/11/2007	458	22 (18.6 : 26.5)	325	247	500	5	60	0		ND	ND	ND	ND	ND	ND	ND	461
04/12/2007	549	26 (22.5 : 29.7)	300	252	500	5	63	ND		ND	ND	ND	ND	ND	ND	ND	485
07/12/2007	640	31 (28.1 : 33.7)	225	170	500	5	53	ND		ND	ND	ND	ND	ND	ND	ND	495
10/11/2007	731	32 (30.1 : 34.9)	200	119	500	5	50	ND		ND	ND	ND	ND	ND	ND	ND	504
01/17/2008	829	33 (31.4 : 35.7)	200	151	500	5	43	ND		ND	ND	ND	ND	ND	ND	ND	520
01/31/2008	843	ND	200	110	500	5	30	1A/1R	-VE	13	39	62	33	45	3.2	51	530
02/05/2008	848	ND	250	132	2000	30	42.5			1	12	23	9	16	4.62		495
03/04/2008	875	ND	250	144	1500	100*	20	1A/1R	-VE	14	22	50	39	33	5.5	57.2	492
03/27/2008	895	35 (33.5–36.9)	250	137	2000	10		ND		ND	ND	ND	ND	ND	ND	ND	495
04/07/2008	910	ND	300	172	2000	10	32	0	-VE	7	28	50	28	39	2.5	61.3	509
04/17/2008	920	29 (26.6–32.7)	300	183	1500	10		ND	ND	ND	ND	ND	ND	ND	ND	ND	497

HTx:heart transplantation, GEP:gene expression profiling, CsA:Cyclosporine-A, MMF:Mofetil Mycophenolate, Pred:Prednisone, LVEF:left ventricular ejection Fraction, AMR:antibody mediated rejection, RAP:right atrial pressure, PCWP:pulmonary capillary wedge pressure, PAS:systolic pulmonary Artery Pressure, PAD:diastolic pulmonary Artery Pressure, PAM:mean pulmonary Artery Pressure, CO:cardiac output, MVO_2_:maximum ventilator oxygen, QTc:corrected QT interval, and ND:not done.

## Abbreviations

HTx: Heart transplantation
GEP: Gene expression profiling
NYHA: New York Heart association
ISHLT: International Society of Heart and Lung Transplantation
QTc: Corrected QT interval
EKG: Electro cardiogram
CsA: Cyclosporine
CO: Cardiac output
HLA: Human leukocyte antigen
PCR: Polymerase chain reaction.
